# Early post-operative thrombosis of the prosthetic mitral valve in patient with heparin-induced thrombocytopenia

**DOI:** 10.1186/1749-8090-7-23

**Published:** 2012-03-13

**Authors:** Attila Cziráki, Zénó Ajtay, Ágnes Nagy, László Márton, Zsófia Verzár, Sándor Szabados

**Affiliations:** 1 Heart Institute, Faculty of Medicine, University of Pécs, Ifjúság u. 13, H-7624 Pécs, Hungary

**Keywords:** artificial mitral valve thrombosis, heparin-induced thrombocytopenia, two-dimensional transesophageal echocardiography

## Abstract

Heparin-induced thrombocytopenia (HIT) is one of the most common immune-mediated adverse drug reactions, with frequencies as high as 2-3% for certain groups of post-cardiac surgery patients. We report on an 50-year-old woman with early post-operative thrombosis of the prosthetic mitral valve due to heparin-induced thrombocytopenia. Non-invasive imaging (two-dimensional transesophageal echocardiography; 2D-TEE) allowed the exact localisation of thrombotic masses and revealed the increase of the mean diastolic mitral gradient. The HIT diagnosis was proved by the clinical scoring system, and with the identification of heparin platelet factor 4-induced antibodies. After the withdrawal of LMWH therapy and the start of intravenous lepirudin treatment, the patient's medical condition improved continuously. Follow-up echocardiography showed a step-wise decrease in the severity of the mean diastolic mitral valve gradient and a complete resolution of thrombus formations. Perhaps we may remind ourselves that, whilst HIT is one of the most common immune-mediated adverse drug reactions for certain groups of post-cardiac surgery patients, it can be managed successfully. We would also stress the importance of serial 2D-TEE examinations in the early post-operative period.

## Background

Patients who are candidates for cardiac surgery require special care in respect of heparin-induced thrombocytopenia (HIT), as this patient population exhibits a relatively high risk for this antibody-mediated, prothrombotic adverse effect of heparin. However, monitoring the platelet count for HIT is a standard feature of post-operative care in cardiac surgery. Unfractionated heparin is remarkably immunogenic, as 25% - 50% of post-cardiac surgery patients develop heparin-dependent antibodies during the 5 - 10 days following surgery [1,2]. Sometimes these antibodies strongly activate platelets and coagulation, thereby causing prothrombotic disorder [3], with the risk of heparin-induced thrombocytopenia at 1% - 3%.

## Case presentation

A 50-year-old housewife with a history of paroxysmal supraventricular tachycardia and rapidly worsened dyspnea due to serious mitral regurgitation was referred to our hospital for the evaluation of valvular disease. The two-dimensional transesophageal echocardiography (2D-TEE) confirmed the diagnosis of severe MR and identified a prolapse of the P2 scallop of the posterior mitral leaflet. Severe MR was confirmed by a *vena contracta *of 7 mm, the effective regurgitant orifice of 0.9 cm^2^, and systolic reversal during Doppler investigation of the pulmonary venous flow. Because of the rapid progression of symptoms (orthopnea and threatening pulmonary edema) the patient was referred to the Cardio-surgery Department, where a prosthetic mitral valve was implanted (Sorin Allcarbon 27). During the first post-operative week the patient was on low-molecular-weight heparin (LMWH) treatment and she was examined by transthoracic echocardiography (TTE) every day.

**Figure 1:**
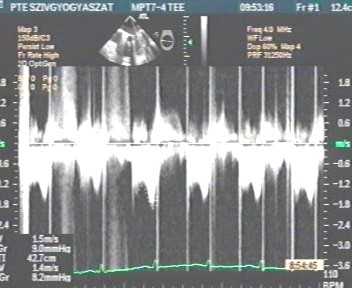
**Transmitral continuous-wave Doppler showing abnormal hemodynamics of the prosthetic mitral valve**. 2D-TEE confirmed the increase of the mean diastolic mitral gradient of 9 mmHg.

**Figure 2:**
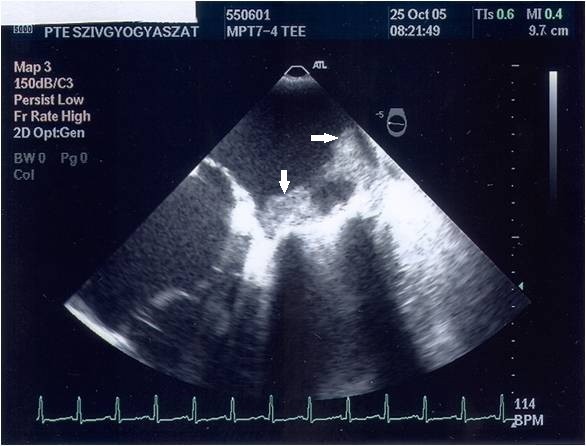
**Mid-esophageal transverse 2-chamber view showing diffuse thrombosis of the prosthetic mitral valve leaflets causing severe leaflet restriction and stenosis**. 2D-TEE revealing large, obstructive thrombotic masses which are attached all around the ring of the prosthetic mitral valve, and adhering to the left atrial wall (double white arrows).

**Figure 3:**
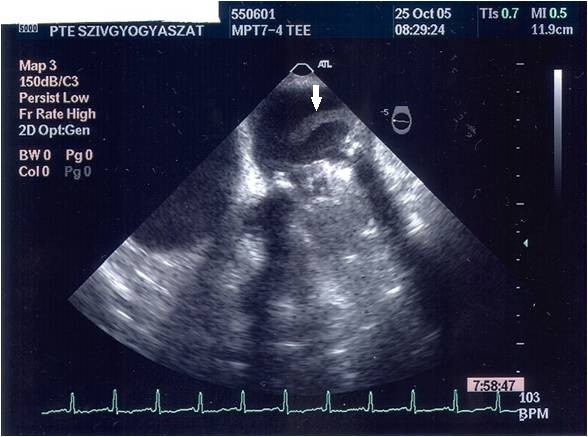
**2-D TEE was performed on the 8^th ^post-operative day**. A 2D-TEE image reveals a 12 mm- long mobile, elongated thrombus which is fixed to the posterior leaflet of the prosthetic valve and floats along the postero-lateral wall to the left atrium (marked with a white arrow).

**Figure 4:**
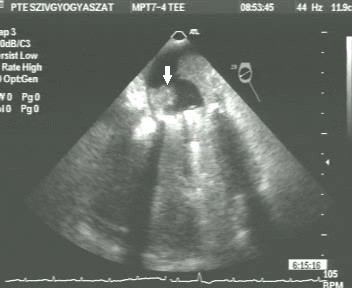
**2-D TEE was performed on the 8^th ^post-operative day**. Another spherical thrombus, 10 mm in diameter and obstructing the proper movement of the anterior leaflet of the prosthetic valve (marked with white arrow).

On the 8^th ^post-operative day, whilst the patient was still on intravenous heparin, the TTE showed a considerable increase of the mean diastolic mitral gradient of 8.9 mmHg. A 2D-TEE was carried out immediately; this confirmed the increase of the mean diastolic mitral gradient (9 mmHg; Figure 1) and completely revealed the underlying mechanisms. We observed an extremely serious obstructive thrombotic mass which was attached to and around the ring of the prosthetic mitral valve (Figure 2). Further, a 12 mm-long mobile, elongated thrombus formation was attached to the posterior leaflet of the prosthetic valve (marked with an arrow on Figure 3). Another spherical thrombus which was 10 mm in diameter hampered the proper movement of the anterior leaflet of the artificial valve (Figure 4). According to the recent guidelines it is well known, that the size and mobility of vegetations are powerful echocardiographic predictors of new embolic events. Patients with vegetations greater than 10 mm are at higher risk of embolism. This risk is even higher in patients with very large (> 15 mm) and mobile vegetations [4].

At the same time we observed a rapid decrease in the platelet count from 178 G/l to 17 G/l. On the basis of the clinical symptoms, we used the '4 Ts' routine blood chemistry pre-test clinical scoring system to test for the possibility of HIT, and proved the high probability of heparin-induced thrombocytopenia [5]. Consecutive anti-PF4 antibodies (Enzyme-Linked ImmunoSorbent Assay HPIA is Heparin Platelet Factor 4-Induced Antibodies [ELISA] Asserachrom HPIA Stago, Asnières France) were positive (optical density > 2), which confirmed the diagnosis of heparin-induced thrombocytopenia [6,7].Anticoagulation with LMWH was immediately stopped and, after initial intravenous lepirudin bolus, continuous iv. infusion was started and maintained for an entire week. Thereafter, for five days combined lepirudin and warfarin therapy was applied until we reached the appropriate INR ratio [2]. As a result of this treatment, the patient's platelet count returned to the preoperative level (213 G/l).

**Figure 5:**
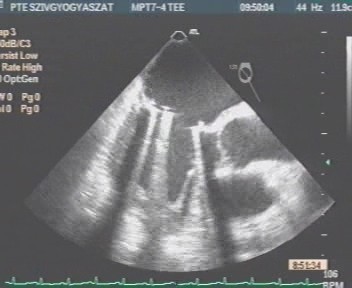
**2D-TEE performed 12 weeks post-operatively**. Complete resolution of the thrombus formations with normal leaflet appearance of the prosthetic mitral valve. (arrows). (This was accompanied by normal artificial valve function with a mean diastolic mitral gradient of 3.8 mmHg.).

A repeat TEE on the 20th post-operative day showed improved artificial valve motion and less thrombotic deposition. A control TEE examination (performed 12 weeks after the operation in the outpatient department) showed a normal artificial valve function with a mean diastolic mitral gradient of 3.8 mmHg and a complete resolution of thrombus formation. Figure 5).

## Conclusions

We present a case of early post-operative thrombosis of the prosthetic mitral valve in a patient with heparin-induced thrombocytopenia. We may remind ourselves that HIT is one of the most common immune-mediated adverse drug reactions, with frequencies as high as 2-3% for certain groups of post-cardiac surgery patients [8,9]. Our case report is a good example that serious complications of HIT, such as early prosthetic mitral valve thrombosis, can be successfully managed by means of the clinical scoring system, with the identification of heparin platelet factor 4-induced antibodies and the timely initiation of appropriate drug treatment. We also emphasise the important role of repeated 2D-TEE examinations in the early post-operative period of cardiac surgery patients. Echocardiography also must be used for follow-up of patients with IE under antibiotic therapy, along with clinical follow-up. The number, type, and timing of repeat examinations depend on the clinical presentation, the type of microorganism, and the initial echographic findings [10]. Weekly TTE may be sufficient in non-complicated streptococcal native IE, while more frequent TEE and TTE controls can be necessary in postoperative staphylococcal early PVE.

## Authors' contributions

In the following we specify the individual contributions of authors to the manuscript. The mitral valve replacement and all surgical procedures were performed by SSz. AC and ZA carried out the TTE and TEE examinations, and prepared the first draft of the manuscript. ZsV prepared the manuscript and managed the perioperative period of the patient. ÁN and LM carried out the ELIZA, which confirmed the diagnosis of HIT and managed the patient until haematologic recovery. All authors read and approved the final manuscript.

## Consent

Written informed consent was obtained from the patient for publication of this Case report and any accompanying images. A copy of the written consent is available for review by the Editor-in-Chief of this journal.

## Conflict of interest

The authors declare that they have no competing interests.
